# Turnover intention and job fit among nurses in Ghana: Does psychological climate matter?

**DOI:** 10.1002/nop2.240

**Published:** 2019-02-20

**Authors:** Julius Atitsogbui, Kwesi Amponsah‐Tawiah

**Affiliations:** ^1^ Department of Organisational and Human Resource Management University of Ghana Business School Accra Ghana

**Keywords:** Ghana, nurses, person‐job fit, psychological climate, turnover intention

## Abstract

**Aim:**

The study examined the relationship between turnover intention and job fit among Registered Nurses in Ghana. Further analysis was done to explore how nurses' psychological climate has an impact on the relationship between job fit and turnover intention.

**Design:**

The study adopted the quantitative research approach and the cross‐sectional survey design in collecting data on the variables of interest.

**Methods:**

Purposive sampling was used to select the eight hospitals from both the public and the private sectors. In all, 322 Registered Nurses participated in the study and the data were analysed using simple and hierarchical multiple regression.

**Results:**

The results of the study showed no statistically significant relationship between nurses' turnover intention and job fit. However, psychological climate was found to fully mediate the relationship between turnover intention and job fit among the participants studied. Finally, the finding adds to theory by proposing a review and an extension of the Attraction‐Selection‐Attrition theory by Schneider (Personnel Psychology, 40, 1987, 437)

## 1 INTRODUCTION

Employees' turnover intention remains a global challenge faced by most organizations particularly in the nursing profession. Despite the several efforts by most organizations and international bodies in addressing this phenomenon, the global deficit for nurses remains alarming (Aiken et al., 2010) and projected to get worst due to the ageing population of nurses (WHO, 2006). The World Health Organization (2013) report estimated 12.9 million global shortage of nurses by 2035, while the Bureau of Labor Statistics also predicts a shortfall of 1.05 million nurses by 2022. What is so intriguing is not the statistics on the nurse deficit which is partly attributed to nurses' turnover, but the cost associated with their turnover. For instance, according to the Nursing Solution Incorporation, (2016) report, an average cost associated with turnover for Registered Nurses ranges between $37,700–58,400 resulting in the average hospital losing about $5.2 M–8.1 M annually. Although the global health labour force crisis is immense and universal, its effects prove to be certainly worse in the sub‐Saharan Africa of which Ghana is not exempted Amediku (2008). This is because out of the 57 countries reported to have a critical shortage of nurses, 36 of them are found to be in the African continent (WHO, 2010). Moreover, WHO (2006) report indicates that, although Africa suffers about 25% of the disease in the world, it has barely 3% and 1% of the world's health workforce and economic resources, respectively.

In Ghana, although this phenomenon has not been extensively studied, earlier studies on nurses' attrition by Zachary (2001) revealed that in 2000, 500 nurses and midwives left Ghana to developed countries to seek for greener pastures which statistically significant accounted for about a double of the number of graduate nurses and midwives who passed out from training in that year. Interestingly, a current study by Boafo (2016) observed that 48.9% of potential Ghanaian nurses under training purposed to leave the country for better prospects elsewhere after their training. This partly contributes to the high nurse–patient ratio since most of the young trained Ghanaian Registered Nurses leave the country for greener pastures in the face of high retiring number of senior nurses. It was not surprising that the nurse‐to‐patient ratio marginally worsened from one nurse to 1,240 patients in 2011–1,251 in 2012 which was above the country's set target of 1–900 patients (GHS, 2013).

The term turnover is generally explained as the mobility of individuals across the limits of an organization (Price, 2001) and has negative effects on a country's nursing shortage (Stanz & Greyling, 2010). It is also conceptualized in different forms, such as organizational turnover (Rothrauff et al., 2011), occupational turnover (Salminen, 2012) and internal turnover (Baumann 2010), voluntary and involuntary (Aneil & Gretchen, 2002). However, turnover intention, which signifies a worker's voluntary and conscious decision to exit an organization or job (Matz, Woo & Kim, 2014), has been shown to be the best predictor and proxy for actual turnover (Juhdi, Pa'wan, & Hansaram, 2013). Cho and Lewis (2012) added that turnover intention is very statistically significant not only as a predictor of actual turnover but also as a signal of employees who may not contribute to an organization at their full potentials. In this paper, turnover intention is conceptualized as an intellectual deliberation process where a Ghanaian Registered nurse has discretionary plans to exit the profession as a result of misfit to the job.

Although factors responsible for turnover intention are inconclusive and inconsistent particularly in management and organization literature, person‐job fit (P‐J fit) is one of the hidden determinants of employees' turnover intention (Memon, Salleh, Harun, Rashid, & Bakar, 2014; Zhang, Yan, Wang, & Li, 2017). It is one of the fit theories of the person–environment theory which emphasizes the congruity between the abilities of an individual worker and that of the demands of the profession. The concept basically suggests an interaction between the individual worker and the job influenced outcomes for both the worker and the firm (Lewin, 1935). In the area of person–environment fit, Kristof Brown, Zimmerman, and Johnson (2005) conceptualized person‐job fit as the abilities, knowledge and skills possessed by an employee to meet the expectations, needs and demands of a job. Ehrhart (2006) further opined that a strong person‐job fit means an individual worker has the requisite skills, the knowledge or competencies to complete the job in question, whereas a poor person‐job fit implies that employees' knowledge, preferences or abilities are not in line with the demands of the job. Evidently, poor person‐job fit has an impact on workplace behaviour. For instance, Boon, Hartog, Boselie, and Paauwe, (2011) and Christensen and Wright (2011) observed that poor person‐job fit leads to job dissatisfaction, low organizational commitment and high turnover intention. Conversely, P‐J fit has been reported to be an important predictor of positive work attitudes and behaviour such as job satisfaction, quality of work life, positive adjustment in new organizations, organizational citizenship behaviour, task performance, dedication, organizational identification, contextual performance, agreement to take the job offer or to decline or exit (Cable & DeRue, 2002; Cable & Edwards, 2004; Guan, Deng, Bond, Chen, & Chan, 2010; Greguras & Diefendorff, 2009; Kristof Brown et al., 2005; Lauver & Kristof‐Brown, 2001; Saks & Ashforth, 2002). Although studies conducted in the developed countries found a positive relationship between P‐J fit and other work‐related attributes as mentioned above, this construct is yet to receive extensive empirical investigations in the nursing profession (Aiken et al., 2010).

Furthermore, psychological climate that refers to the individual employee's held perceptions and interpretation of the work environment has been an area of interest for most scholars and practitioners due to its influence on other individual and organizational outcomes. However, over the years, scholars have focused on how organizational climate has an impact on individual attributes and organizational outcomes (Al‐Khasawneh, 2013; Jeswani & Dave, 2012; Sökmen & Şahingöz, 2017) while little is known about the influence of the individual employee's psychological climate. Meanwhile, earlier scholars such as Lewin (1935) opined that a person's behaviour is systematically influenced by the psychological field where they exist. Holt (1993) added that an employee's perception of the workplace environment has a greater effect on the actions of that employee than the person's individual qualities. Mobley (1982) in his socio‐psychological framework recons that a workers' decision to leave an organization or job is a response to the discrepancies between psychological climate and the expectations, needs and aspirations of the employee. It can, therefore, be deduced from the above assertions that an employees' psychological climate of the work environment influences their intention to stay or leave an organization or job. Related to the above premise is that most of the studies in this area only focused on the direct relationship between person‐job fit and turnover intention (Aziri, 2011; Harpert, 2012; Zhang et al., 2017), while other possible confounding mechanisms are yet to be investigated. It is against this background that the current paper argues that nurses who score high on P‐J fit are more likely to develop a positive psychological climate of their work environment which may invariably reduce their intention to exit. Therefore, this current paper explores how person‐job fit predicts turnover intention as well as how psychological climate mediates this relationship among Registered Nurses in Ghana.

## 2 THEORETICAL FRAMEWORK

The study is underpinned by the Selection‐Attraction‐Attrition theory (Schneider, 1987) which proposes that “people in any organisation are unique in that they are the ones attracted to, chosen by and choose to remain with an organisation.” Besides, the Theory of Work Adjustment to turnover (TWA) (Dawis & Lofquist, 1984) is used in examining the interaction between an individual worker and the work situation. TWA is mainly concerned with “adjustment” to the expectations and rewards of work. Here, the personnel and the work environment are considered to have a joint relationship which mutually influences the tenure of work.

### 2.1 Person‐job fit and turnover intention

There is considerable evidence that a high level of P‐J fit has several positive outcomes. In management literature, empirical investigations have shown that individual workers who possess a great degree of job fit have higher degrees of job satisfaction, organizational commitment, organizational identification, perceived organizational support, increased work performance, reduced turnover intentions and actual turnover (Bhat, 2014; Brkich, Jeffs, & Carless, 2002; Kristof, 1996). Other scholars have demonstrated that person‐job fit could be used as an effective tool for hiring and selection of employees (Sekiguchi & Hiber, 2011). Although there is no universal agreement on the determinants of turnover intention, person‐job fit is demonstrated to have highly correlated with intention to leave. For instance, Leng and Chin (2016) did a study among employees in a marketing department to examine how P‐J fit, personality and organizational commitment have an impact on intention to continue working with an organization. Generally, the results revealed a statistically significant relationship between the three variables mentioned. However, organizational commitment partially mediated P‐J fit and intention to continue working with an organization. Thus, as organizational commitment increased, intention to continue working with an organization likewise increased. This implies that, when an employees' knowledge, skills and abilities fit the requirements of the work, or when his or her beliefs and values are similar to that of the organization, their commitment upsurges and they are less likely to resign from the profession.

On the contrary, Chang, Chi and Chuang (2010) observed a positive relationship between person‐job fit and turnover intention. Their results suggest that a worker whose knowledge, expertise and capabilities match the demands of the job is more probable to locate alternatives elsewhere which may surge their perceived job mobility and hence increase intention to exit. Similarly, Ilyas (2013) adopted variables such as person‐job fit, organizational commitment, job satisfaction and intention to exit in examining organizational behaviour. He conjectured that person‐job fit will be positively linked to job satisfaction and negatively linked with turnover intention. While organizational commitment was also found to have influenced the relationship between person‐job fit and intent to quit, person‐job fit rather showed a positive relationship with turnover intention (*r* = 0.017) but was not statistically significant supported by the regression analysis.

In addition, scholars opined that P‐J fit is distinct from P‐O fit and should be treated separately in an analysis (Kristof, 1996) and in most occasions uncorrelated when examined concurrently (Lauver & Kristof‐Brown, 2001). Due to the inconsistency with regard to the relationship between P‐J fit and turnover intention as reported by the above studies, the current paper proposes that nurses who fit their job will be less likely to exit the nursing profession. Hence, hypothesized that,

H1: There will be a statistically significant negative relationship between person‐job fit and turnover intention among registered Ghanaian nurses.

### 2.2 Psychological climate as a mediator

Psychological climate has recently been an area of interest for both scholars and practitioners due to its influence on other individual and organizational outcomes. Evidently, this construct has relatively been investigated in the academic field (Biswas, 2010), particularly in relation to turnover intention (Balogun, Adetula, & Olowodunoye, 2013). However, most of the relevant and available studies explored its direct effect on other organizational variables (Balogun et al., 2013; Biswas, 2010; Sahin, 2011). In terms of indirect relationships, few studies like Hassan, Akram, and Naz (2012) and Sahin (2011) are advancing in the areas of psychological climate, job fit and turnover intention. However, the findings have been inconsistent and inconclusive. For example, while the findings of Sahin (2011) showed a partial mediation of affective commitment on the relationship between psychological climate and turnover intention, Langkamer and Ervin (2008) recorded a full mediation. Similarly, a study by Hassan et al. (2012) among bankers in Pakistan indicates an inverse relationship between person‐job fit and turnover intention. However, the meditational analysis of psychological climate revealed a fully indirect effect on the relationship between turnover intention and P‐J fit. Although available literature and meta‐analysis (Biswas, 2010) suggest a relationship between person‐job fit and turnover intention, some meta‐analytical results showed a weak relationship between these two constructs (Kristof Brown et al., 2005; Zang et al., 2017). It is against this background that the current study proposes psychological climate as one mechanism through which person‐job fit will have an impact on turnover among registered Ghanaian nurses.

H2: Psychological climate will mediate the relationship between Person‐Job Fit and turnover intentions among registered Ghanaian nurses

## 3 METHOD

### 3.1 Research approach and design

The study employed a quantitative research approach to test for the relationship between the various variables under study. This allows for gathering of data and information which are quantifiable and can be subjected to statistical treatment for the purposes of confirming or refuting an alternate knowledge claim (Creswell, 2003). The cross‐sectional survey design was used in data collection as it allows the researcher to gather large sample of the target population data at one point in time through the use of questionnaire.

### 3.2 Participants

The study purposively and conveniently sampled 322 Registered Nurses from eight Ghanaian hospitals in the Greater Accra Region on their turnover intention, job fit and psychological climate levels. The hospitals included the Greater Accra regional hospital (Ridge), Lekman Hospital, Police Hospital, Legon Hospital, Family Health Hospital, Manna Mission Hospital, Lister Hospital and Pentecost Hospital, Ghana.

### 3.3 Measures

Person‐job fit was measured using Cable and DeRue's (2002) six‐item scale. It is scored on a five‐point Likert scale that ranges from 1 = strongly disagree–5 = strongly agree with a composite Cronbach's alpha of 0.73. One of the items of the scale includes “there is a good fit between what my job offers me and what I am looking for in a job.” The nurse turnover intention scale (NTIS) developed by Roodt (2004) was also used to collect data regarding nurses' turnover intention. The items are scored on a seven‐point Likert scale that ranges from 1 = mostly not often–7 = mostly often with a Cronbach's alpha of 0.84. It is a 14‐item scale and has one of the items as, how often have you considered leaving your current job. The psychological climate scale, which consisted of 21 items, was adapted from Brown and Leigh (1996). The items are scored on a five‐point Likert scale that ranges from 1 = strongly disagree–5 = strongly agree with Cronbach's alpha of 0.84. The scale has six subscales consisting of supportive management, recognition, self‐expression, role clarity, contribution and challenge. One of the items of the scale includes, my boss is flexible about how I accomplish my job objectives etc.

### 3.4 Ethical approval

The researchers ensured ethical consideration by allowing the individuals nurses and the various institution to participate in the study at will. Cover letters containing the research topic, name of researchers and their intention to conduct the study in the various hospitals were presented to the administrators of those facilities (Appendix A). This was done to secure permission and to avoid coercion and manipulations of study institutions and participants. The nurses' rights to self‐determination were ensured since they decided independently to whether to be part of the study without any coercion. They also had the right not to answer any questions that will cause discomfort and to disclose or not to disclose personal information as well as to ask for clarification about any aspect that posed some uncertainty. The researcher also fully disclosed the nature of the study to the respondents and alerted them about their rights to participate or to refuse to participate in the study.

**Figure 1:**
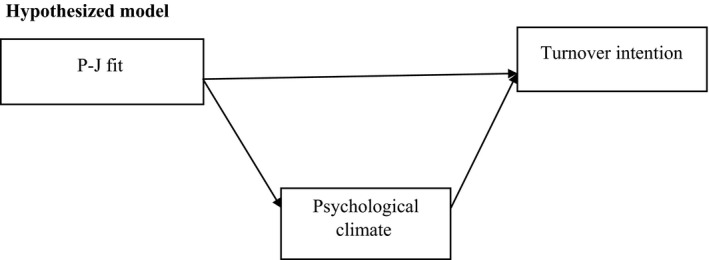
A pictorial representation of the hypothesized relationship between the predictor variables and the criterion variable

**Figure 2:**
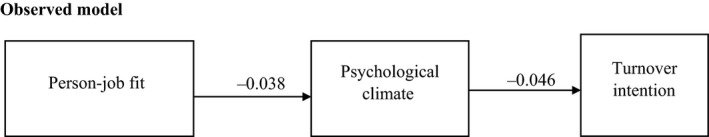
Observed model showing the relationship between the predictor variable, mediator and the criterion variable

From Table 1, it can be observed that the gap between female and male nurses stood at 49.6% in favour of females. The males constitute 25.2% with female making up for the remaining 74.8%. This simply shows that most respondents were female. Regarding the level of education, most respondents had attained a diploma in nursing representing 72.7%. This may be due to the fact that, in Ghana, a Registered nurse must have successfully completed 3 years nursing training course and passed his or her licensor examinations. However, 21.4% attained first‐degree qualification, 4.3% master's degree and 1.6% had other health qualifications. As observed, most of the respondents were general nurses, followed by midwives representing 47.5% and 18.6%, respectively. However, out of the entire sample eye and ear nurses were quite few representing 7.5% and 7.5%, respectively. The high figure as shown by general nurses might be as a result of the high intake of general nurses during admission. Moreover, most nurses enter the profession as general nurses before veering into their respective specializations (Figures 1 and 2).

**Table 1:** Demographic characteristics of participants

Variable	Frequency	Percentage
Gender		
Male	241	74.8
Female	81	25.2
Category of nurses		
General nurses	153	47.5
Midwives	60	18.6
Eyes nurses	24	7.5
Ear nurses	24	7.5
Psychiatric nurses	38	11.8
Others	23	7.1
Educational level		
Diploma	234	72.7
Degree	69	21.4
Masters	14	4.3
Others	5	1.6
Type of hospital		
Public	188	58.4
Private	134	41.6
Tenure		
1–5	220	68.6
6–10	71	22
11–15	15	4.4
16 and above years	16	5.0
Terms of employment		
Permanent worker	55	17.1
Shift worker	69	21.4
Casual worker	198	61.5

## 4 HYPOTHESES TESTING

The first hypothesis was analysed using simple regression to examine the relationship between the predictor and the criterion variables. Similarly, hierarchical multiple regression analysis following the Baron and Kenny's (1986) approach was used in examining the mediation effect of psychological climate on the relationship between person‐job fit and turnover intention among registered Ghanaian nurses.

H1: There will be a statistically significant negative relationship between Person‐Job Fit and turnover intention among registered Ghanaian nurses.

The result from Table 2 shows no statistically significant relationship between person‐job fit and turnover intention as observed by the model (*β* = 0.038, *p *> 0.05). This implies that the nurse's fit to the job in that their knowledge, skills and abilities match with the expectations and demands of the profession; does not necessarily predict their retention on the job. In other words, fitting the job does not solely or necessarily foretell nurses' retention which in effect proposes other factors other than P‐J fit may influence nurses' turnover intention in Ghana.

**Table 2:** Summary of simple linear regression analysis for relationship between person‐job fit and turnover intention

Model	Unstandardized coefficients		Standardized coefficients	Sig.
	*B*	Std. error	Beta	
2				
Constant	60.066	3.341		0.000
Person‐job fit	−0.078	0.115	−0.038	0.495

Note

Dependent variable: Turnover intention, *R* ^2^ = 0.001, *p* > 0.

Therefore, the hypothesis that “there will be a statistically significant negative relationship between person‐job fit and turnover intention was also not confirmed by the current study".

H2: Psychological climate will mediate the relationship between person‐job fit and turnover intentions of registered Ghanaian nurses.

Table 3 shows the mediation effect of psychological climate on the relationship between person‐Job fit and turnover intention. To test for the above‐mentioned hypothesis, four steps were employed. In the first step, the predictor (P‐J fit) was regressed on TI and was observed not to be statistically significant (*β* = −0.038, *p *> 0.05). Though the procedures recommended by Baron and Kenny (1986) require a statistically significant relationship between the predictor variable and the criterion as a condition for mediation analysis, other scholars have argued that such a condition is not crucial (Hayes, 2009; Rucker, Preacher, Tormala, & Petty, 2011). Hence, the researchers proceeded to the next stage. In the second stage, a statistically significant positive effect was observed when person‐job fit was regressed on the mediator variable as observed (*β* = 0.313, *p *< 0.001). In the third step, the mediator (psychological climate) was regressed on the criterion (turnover intention) and was found to be statistically significant (*β* = −0.144, *p *< 0.01). To test for the indirect effect (path a × path b) in step four, the researchers multiplied the standardized coefficients of the predictor–mediator relations (path a) and the mediator–criterion relations (path b) and found it to be higher (indirect effect = −0.046) than the direct effect of predictor on the criterion (path c; *β* = −0.038, *p *> 0.05).

**Table 3:** Summary of mediating effect of psychological climate on the relationship between person‐job fit and turnover intention

Step	Predictor	Criterion	*B*	*SE*	*β*
1	P‐J fit	T. Intentions	−0.078	0.115	−0.038
2	P‐J fit	P. Climate	0.425	0.072	0.313***
3	Climate	T. Intentions	−0.218	0.084	−0.144**
4	P‐J fit→Climate	T. Intention		−0.046	

Note

Statistically significant indirect effect (Boot *SE* = 0.023, LLCI = −0.100, ULCI = −0.008).

**p < .01, ***p < .001

To test for statistically significant mediation, the bootstrapping method as described by Hayes (2009) was used with a bootstrapped sample of 5,000. The results of the bootstrap showed boot standard error of −0.023 which fell within the negative lower and upper confidence interval of −0.100 and −0.008. Both lower and upper confidence interval falling in the same direction signifies a statistically significant full mediation effect. This is because the predictor on its own could not statistically significant reduce turnover intention but rather did so when psychological climate was present. This shows that psychological climate fully transmitted the effect of person‐job fit on turnover intentions. Therefore, the hypothesis that “psychological climate will mediate the relationship between person‐job fit and turnover intention” was supported by the present study.

## 5 DISCUSSION

This paper sought to examine whether person‐job fit could predict turnover intention among registered Ghanaian nurses and how psychological climate has an impact on this relationship. The finding of the study showed that person‐job fit could not statistically significant predict turnover intention among registered Ghanaian nurses. This implies that having an agreement between the nurses' job descriptions and knowledge, expertise, capabilities and other features (personality, temperament etc.) does not necessarily predict nurses' retention in the profession. In other words, the nurses were indifferent about their intention to leave even when they find congruence between their KSAs and the demands of the job. The absence of alternative jobs coupled with the current prevailing high rate of unemployment in Ghana may partly account for the reason why the nurses were indifference about intention to leave the profession (Amankwaa & Anku‐Tsede 2015). According to Shah, Deen and Szabist (2015), turnover intention rates are usually high where the unemployment rate is low and more attractive alternative opportunities are available. Although this finding did not support the usual position established in extant literature (Shah et al., 2015; Guan et al., 2010; Hassan et al., 2012; Leng & Chin 2016) that high person‐job fit leads to lower turnover intention, it was, however, consistent with findings of Ilyas, (2013) and Chang, Chi & Chuang (2010).

In addition, the result of the current paper emphasized the position of the work adjustment to turnover intention proposed by Dawis and Lofquist (1984). The theory holds that workers together with the work environment have a joint relationship which mutually affects the tenure of work. In other words, a correspondence is achieved when an employee brings certain skills and abilities into the work environment (demand‐ability) and the environment also fulfils the requirements of the individual employee (need‐supply). The fit between the nurse and their job as observed by the current data means that it is possible the periodic training sessions, conferences and short courses organized by the Ghana Health Service contribute to their ability to meet the demand of their job. Putting the findings in the light of the TWA theory, it is observed that most of the nurses have been in practice for over a year and should have in the given space of time, practice and training, adjusted to the demands of the job. Taking cognizance of the huge investment made in terms of their years spent in school and training in specific fields of practice (e.g., eye nurse), it will be detrimental on the part of a nurse to consider leaving the profession in the absence of readily available alternative jobs hence the observed indifference to turnover intention.

The second objective of the study proposed psychological climate as a mechanism through which person‐job fit will influence turnover intentions. The analysis of the hierarchical multiple regressions showed that psychological climate statistically significant and fully mediates the relationship between person‐job fit and turnover intention among Ghanaian Registered Nurses. This implies that the predictor (person‐job fit) could not on its own statistically significant reduce turnover intention but rather did so in the presence of psychological climate. In effect, the individual nurse's psychological climate that refers to the nurse's perception and interpretation of work environment fully transmitted the effect of person‐job fit on turnover intention. Thus, when a nurse fits the job, he or she becomes more psychologically attached or engaged with the job through their positive perception and interpretation of the work environment leading to a reduction in turnover intention. The result of the study emphasizes psychological climate as an important tool in nurses' retention. This reiterates the fact that a nurse can have all the needed skills, abilities and knowledge in meeting the demands of the job but can, however, decide to leave the job probably because of his or her perception, experiences and interpretation of leadership style, relationship between co‐workers, conditions of service and other factors in the work environment. On the other hand, when an employee has a positive psychological climate, thus having supportive management, being recognized for work done as well as working in an environment where duties are clearly defined, it indirectly leads to and reduces his or her intention to leave the job.

The above finding is in line with that of Hassan et al. (2012) who found psychological climate to have fully mediated the relationship between person‐job fit and turnover among Bankers in Pakistan. This means that to reduce turnover intention among employees, management must not consider their fitness to the job alone but must also focus on creating an environment that will positively influence the psychological climate of the workers.

## 6 CONTRIBUTIONS, RECOMMENDATIONS AND CONCLUSION

The primary strength of the current paper lies in its ability to contribute to an ever‐increasing academic discourse on turnover intention by examining the relationship between P‐J fit and turnover intention among Registered Nurses in Ghana.

Theoretically, the results of the paper propose that other factors other than P‐J fit can influence the turnover intentions of employees. Specifically, psychological climate was found to have statistically significant accounted for a reduction in variance of registered Ghanaian nurses' intention to leave the job. Contrary to Schneider's ASA theory, the current paper showed that P‐J fit does not necessarily lead to retention. However, the individual's perception of the work environment in terms of trust dynamism and other affect laden dimension was crucial for the nurses' continuous stay in their job and organizations. This suggests that the ASA theory should be re‐examined to include other factors such as psychological climate in determining employees' retention.

The current paper demonstrates the significance of psychological climate in contemporary organizations particularly in healthcare management. It offers additional insight into the management of nurses in both private and public health facilities highlighting the role of psychological climate and person‐job fit in the management of nurses' turnover behaviour. Managers of healthcare practitioners particularly nurses should create work environment that have a positive impact on nurses' behaviours. The various dimensions as included in the psychological construct such as supportive management, recognition, self‐expression and role clarity should be encouraged and inculcated the human resource policies and practices of the organizations.

## 7 LIMITATIONS AND RECOMMENDATION FOR FUTURE STUDY

The present study certainly has some limitations. However, these shortfalls do not undermine the significance of its findings. Notable limitations identified are the use of a cross‐sectional design which can only capture nurses remaining in their jobs and may not be able to track the number nurses who that had a turnover intention and consequently left the job. In addition, using the quantitative research technique to investigate the relationship between the various variables does not allow for deeper understanding and insight into the various variables studied and hence makes difficult to establish cause and effect relationships. Future studies should consider a qualitative or mixed method approach to assess the relationship between the variables. These techniques may give a deeper and clearer understanding of the constructs and as well increase the external validity of the research.

## 8 CONCLUSION

The management of health workers particularly nurses has increasingly become imperative in the face of challenging health‐related issues and organizational dynamisms. Crucial among these challenges needing much attention are the issues characterized with enlisting nurses who have the right dispositions for the work as well as creating necessary workplace atmosphere needed for organizational efficiency and effectiveness. It is in response to this that paper highlights the significance of psychological climate on the turnover intentions of Registered Nurses. The findings of the study suggest that the nurses' positive perceptions and assessments of their work environment thus, the policies, structures and systems of the hospital warrant their continuous stay on the job rather than just their fitness to the job. Hence, in maximizing organization effectiveness and nurses' retention, the HRM policies, systems and programs of the hospitals should be tailored towards creating congenial working atmosphere.

## CONFLICT OF INTEREST

The authors of this paper have no conflicts of interest. They are solely responsible for the content and writing of this paper.

## AUTHOR CONTRIBUTIONS

The authors were involved in the design of the study, data analysis and interpretation. The corresponding author was responsible for data collection and drafting of the manuscript. The second author critically proof read and reviewed the manuscript for publication.
